# Evaluation of gingival condition in asthmatic children

**DOI:** 10.4317/jced.63556

**Published:** 2026-02-26

**Authors:** Andrea Ruiz-Hernández, Francisco Javier Silvestre, Miguel Tortajada-Girbés, Javier Silvestre-Rangil

**Affiliations:** 1 Department of Stomatology, University of Valencia, Valencia, Spain; 2 Chairman. Department of Stomatology, University of Valencia, Spain; 3 Professor. Department of Pediatrics, University of Valencia, Valencia, Spain; 4 Assistant Professor. Department of Stomatology, University of Valencia, Spain

## Abstract

**Background:**

Asthma is considered to be the most common chronic respiratory disorder in children, and it has been suggested that asthmatic children may be at an increased risk of developing gingival disease. A study was made to evaluate the level of oral hygiene and gingival health in asthmatic children, and to explore their possible relationship with the severity of asthma.

**Materials and Methods:**

A total of 187 children (108 with asthma and 79 healthy controls) between 6-16 years of age were studied. A questionnaire was used to collect information on habits of oral hygiene. The presence of mouth breathing was recorded, and an oral clinical examination was carried out based on the following indices: Silness-Löe plaque index, simplified Greene-Vermillion oral hygiene index, Löe-Silness gingival index, Ainamo and Bay bleeding on probing index, and the community periodontal index (CPI). The severity of asthma was classified following the recommendations of the Spanish Guide on the Management of Asthma (Guía Española para el Manejo del Asma [GEMA]).

**Results:**

The level of oral hygiene was similar in both groups. Mouth breathing was more frequent among the asthmatic children (p&lt;0.001), and the gingival index and bleeding on probing scores were higher (p=0.005 and p=0.013, respectively).

**Conclusions:**

Mouth breathing was more common in the asthmatic children, and they had higher gingival index and bleeding on probing scores than the healthy controls, despite good habits of oral hygiene.

## Introduction

Asthma is a chronic inflammatory disorder of the airway characterized by bronchial hyperresponsiveness and variable airflow obstruction that is totally or partially reversible either spontaneously or with treatment. The associated symptoms are wheezing, dyspnea, chest tightness and cough, and their appearance, intensity and frequency vary over time (1 , 2). The World Health Organization (WHO) considers asthma to be the most common chronic respiratory disorder in childhood, affecting approximately 5.5 million children in the European Union (3). Many studies have been published on the condition of the oral cavity in childhood asthma, with contradictory findings. While some authors suggest that asthmatic children have a greater risk of developing gingival disease, other investigators have reported no such association (4 , 5). Poor oral hygiene, with the accumulation of dental plaque, leads to gingivitis, which in turn can evolve towards periodontal disease (6). Davidovi et al. reported deficient oral hygiene together with poorer periodontal tissue health to be more frequent in asthmatic children (6). Hyyppä (7), Kumar et al. (8) and Mehta et al. (9) recorded significantly higher dental plaque and gingival index scores in asthmatic subjects versus a control group. The medications used to treat asthma con reduce salivary secretion. Considering that the protective mechanisms present in saliva balance the interactions between bacteria and the immunological factors that contribute to maintain periodontal health, this decrease in secretion could favor bacterial colonization and dental plaque formation, thereby affecting the severity of periodontal disorders in these patients (6 , 8). The mouth breathing habits often seen in asthmatic patients due to airway obstruction could also reduce the protective properties of saliva and increase the risk of developing periodontitis (6). In the same way that certain chronic disorders such as asthma may be regarded as risk factors for the development of periodontal disease, some recent studies consider that the oral pathogens causing gingivitis and periodontal disease could play an important role in the exacerbation of chronic respiratory disorders, since dental plaque may serve as a reservoir for respiratory pathogens. In this regard, the evidence suggests that the elimination of dental plaque could improve respiratory function in asthmatic children (6). The present study was carried out to evaluate the level of oral hygiene and gingival health in asthmatic children, and to explore their possible relationship with the severity of asthma.

## Materials and Methods

- Study design A cross-sectional study was carried out in children seen in the Departments of Pediatrics and Dentritry of Dr. Peset University Hospital (Valencia, Spain), using non-probability convenience sampling, over a three-year period. The participants and their families were informed about the characteristics of the study, and written informed consent was obtained from the parents and/or tutors. The study was approved by the local Ethics Committee (ref. 42/17). The total sample size required was estimated to ensure that the two proportions in the case and control groups could be detected as significantly different with a statistical power of 80% and a 95% confidence level. A prevalence of positive outcomes of 50% in one group compared to 20% in the other (proportions 0.5 vs. 0.2) was considered relevant and indicative of a potential effect of the pathology. Therefore, the sample had to include at least 180 subjects for the difference to be detected as statistically significant with 80% power. - Study participants Children of either sex and aged 6-16 years were included. The study group consisted of children diagnosed with bronchial asthma for at least one year according to the Spanish Guide on the Management of Asthma (Guía Española para el Manejo del Asma [GEMA]). The control group in turn consisted of healthy children with an age and gender distribution similar to that of the asthmatic patients. Children with any other associated medical condition were excluded, as were those receiving drug treatment other than that prescribed for asthma, or who had undergone antibiotic treatment in the month prior to the exploration. Children receiving orthodontic (fixed or removable) treatment were also excluded. - Study protocol Habits referred to oral hygiene were documented prior to examination of the oral cavity: age at the start of tooth brushing, frequency, duration and supervision of tooth brushing, type of brush, use of toothpaste or oral rinse with fluor and dental floss. Mouth breathing confirmed by the buccodental exploration was also recorded. As regards severity, the children were classified as having mild intermittent or persistent asthma, moderate persistent asthma, or severe persistent asthma, based on the symptoms and the medication required to keep the condition under adequate control, according to the recommendations of the GEMA (2). The clinical examination was carried out in a conventional dental chair, under illumination and using a WHO type periodontal probe and a flat intraoral mirror. Oral hygiene was evaluated using the Silness-Löe plaque index and the simplified Greene-Vermillion oral hygiene index. The gingival condition in turn was assessed based on the Löe-Silness gingival index and the Ainamo and Bay bleeding on probing index. The community periodontal index (CPI) in turn was used to evaluate periodontal health following the recommendations of the WHO for individuals under 15 years of age. The diagnosis of oral breathing habit was performed using the Glatzel mirror. - Statistical analysis The data obtained were analyzed using the Student t-test for independent samples, the chi-square test to assess the homogeneity of categorical variables, multifactor analysis of variance (ANOVA), and binary logistic regression analysis to relate the oral health variables. Statistical significance was considered for p &lt; 0.05.

## Results

A total of 187 children (108 with asthma and 79 healthy controls) between 6-16 years of age were included in the study. The mean age of the children with asthma was 10.3 ± 2.3 years, versus 11.3 ± 2.6 years in the control group. The comparison of means (t-test) evidenced differences between the groups (p=0.008); the statistical analyses were therefore adjusted for age. With regard to gender distribution, 65.75% of the asthmatic children were males, versus 54.4% of the controls - the difference being nonsignificant. While oral hygiene was similar in the two groups, significant differences were observed in relation to mouth breathing (36.1% of the asthmatic patients versus only 5.1% of the controls) (p&lt;0.001). - Oral hygiene, gingival and periodontal indices by groups There were no significant differences between the groups in terms of the Silness-Löe plaque index or simplified Greene-Vermillion oral hygiene index (Table 1), thus evidencing similar oral hygiene conditions in both groups.

Table 1

**Figure 1:**
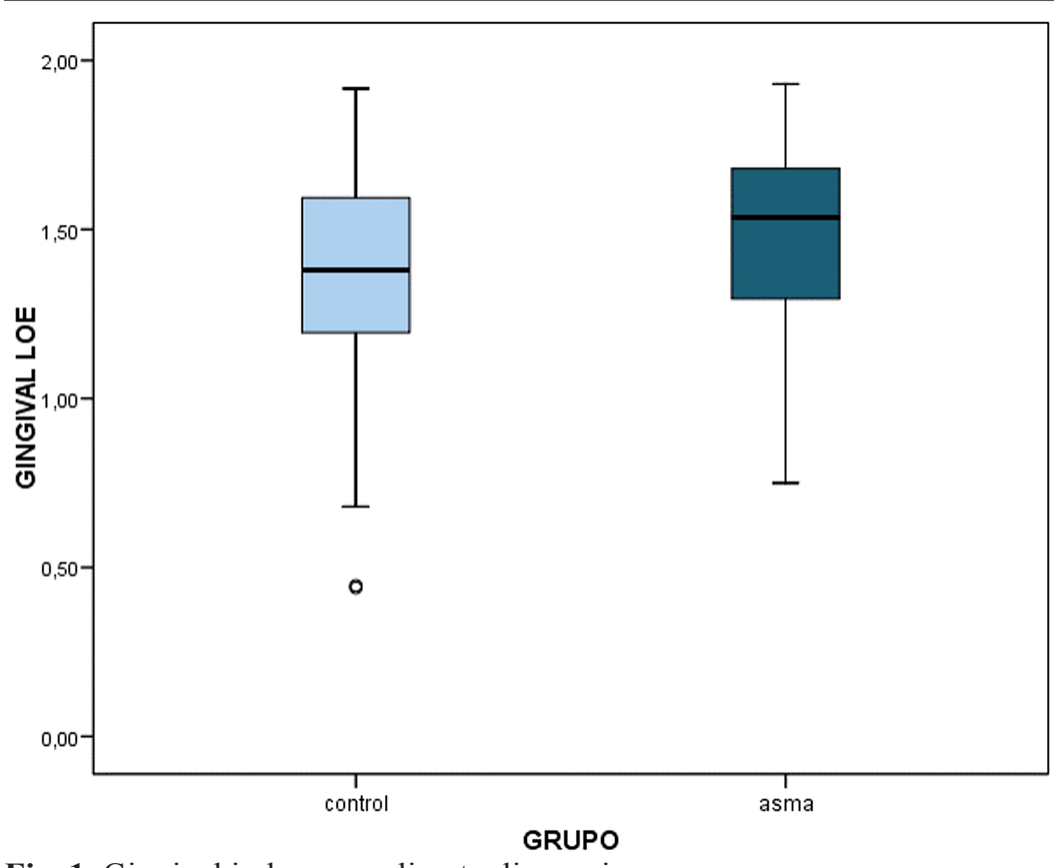
Gingival index according to diagnosis.

The mean Löe-Silness gingival index was 1.48±0.27 in the asthmatic children and 1.36±0.32 in the controls - the difference being statistically significant (p=0.005) (Table 1, Fig. 1).

1

**Figure 2:**
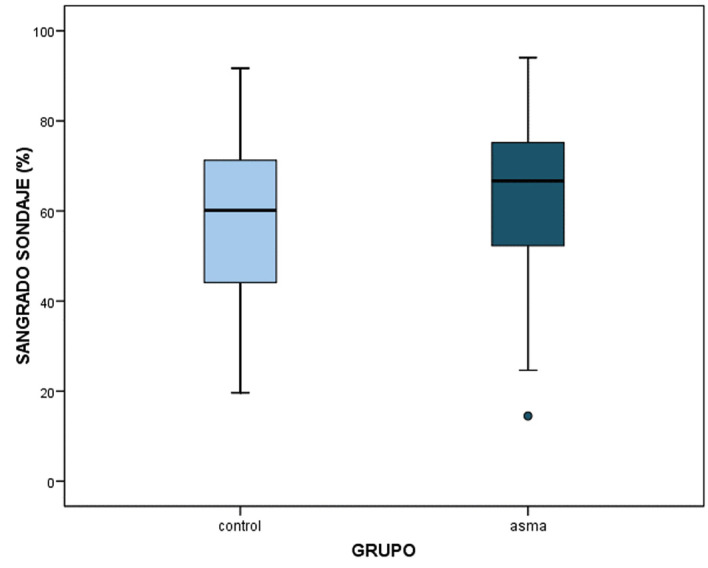
Bleeding on probing index according to diagnosis.

Likewise, significant differences were observed in terms of the Ainamo and Bay bleeding on probing index. The percentage bleeding score in the asthmatic group was 63.76±16.58% versus 57.74±18.10% in the control group (p=0.013) (Table 1, Fig. 2).

2

**Figure 3:**
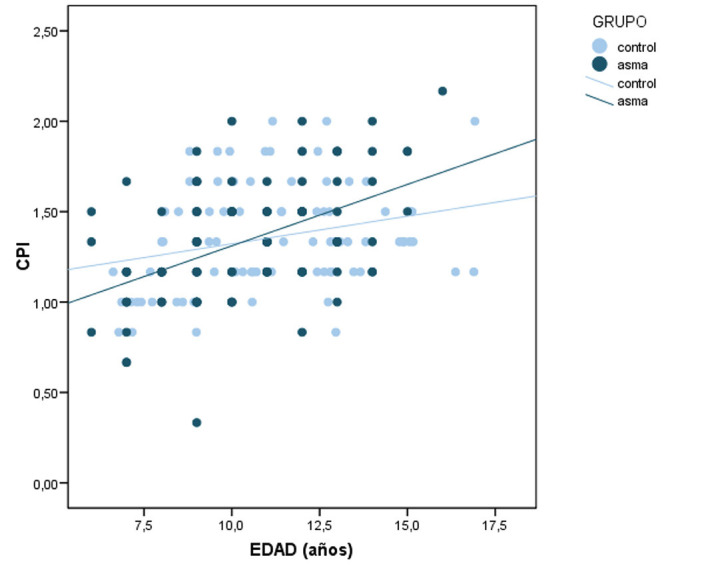
Dispersion plot stratified by group between age and the community periodontal index.

The difference in score of the community periodontal index was not significant between the two groups. The mean CPI score in the asthmatic group was 1.33±0.32 versus 1.36±0.30 in the control group (p=0.780) (Table 1). On the other hand, the mean CPI score was seen to be correlated to the age of the child (F=3,87; p=0.051), with older age being associated to higher mean CPI scores (Fig. 3).

3

- Oral hygiene, gingival and periodontal indices by asthma severity The scores corresponding to the oral hygiene, gingival and periodontal indices showed no significant association to the severity of asthma. Although there were marked differences between the groups in terms of bleeding index, statistical significance was not reached (p=0.070). The mean score was 61.51±16.31% in the patients with mild asthma and 68.07±16.46% in those with moderate-severe asthma (Table 2).

Table 2

## Discussion

The results of the present study showed gingival inflammation to be more common in the group of asthmatic children than among the healthy controls. In contrast, Ryberg et al. (10) and Bjerkeborn et al. (11) did not find asthma disease to have a negative impact upon the gingival condition of asthmatic children. However, other investigators have reported greater gingival alterations in asthmatic patients (9 , 12 , 13). In concordance with other studies (14 , 15), the community periodontal index (CPI) score showed no significant differences between the two groups. Nevertheless, Shashikiran et al. (16) and Davidovi et al. (6) reported a poorer periodontal condition in asthmatic children than in the controls. Some authors consider that one of the reasons why asthmatic children do not have a higher prevalence of periodontal disease is because the latter is more frequently found in older individuals. In this respect, although asthmatic children present greater gingival inflammation, this disorder would not yet manifest as a greater frequency of periodontal disease (6). In line with our findings, Ehsani et al. (17) and Brigic et al. (18) recorded no significant differences in the presence of dental plaque between the groups. Ferrazzano et al. (14) did not observe a greater presence of tartar and plaque in asthmatic children compared with the controls. Indeed, Mazzoleni et al. (19) reported the presence of dental plaque to be lower in asthmatic children, with significant differences versus the control group. In contrast, Kumar et al. (8) and Santos et al. (20) observed greater levels of visible plaque in the asthmatic group, and McDerra et al. (21) reported the amount of tartar and plaque to be significantly greater in asthmatic children than in the controls. Some authors consider that the higher prevalence of tartar in asthmatic children could be due to an increase in the levels of calcium and phosphate in parotid and submaxillary gland saliva (5 , 21). The greater presence of dental plaque and tartar could explain why asthmatic patients present more gingivitis, due among other reasons to the fact that parents tend to focus their attention on asthma care and neglect other aspects such as oral health (5 , 20). However, in our study the oral hygiene questionnaire showed the habits of oral hygiene to be similar in both groups. Nevertheless, the asthmatic children clearly had more gingivitis and bleeding on probing. Our results suggest that the causes underlying greater gingival alterations in the asthmatic children could involve immune factors as well as habits such as mouth breathing (9 , 21). With regard to the role of the immune system in gingival inflammation, Hyyppä (22) found the concentration of IgE to be elevated in the gingival tissue of asthmatic individuals. Previous studies likewise found the salivary IgE concentrations to be increased in both asthmatic adults and asthmatic children (7 , 23). Allergic asthma is associated with an IgE antibody response to different allergens and the development of an inflammatory reaction. The observed increase in IgE levels in saliva and gingival tissue therefore could be related to gingivitis and periodontal disease (7 , 23 , 24). Likewise, asthma and periodontal disease could have similar pathophysiological characteristics. Thus, for example, it has been shown that certain cytokines such as IL-5 and IL-6, which participate in inflammatory processes of the respiratory tract mucosa, are also present in inflamed periodontal tissues (9). In line with our own observations, Stensson et al. (25) found mouth breathing to be significantly more common in asthmatic patients. In turn, other authors have reported that mouth breathers have a greater presence of gingivitis in the anterior maxillary zone. In this sense, mouth breathing could result in mucosal dehydration and the induction of gingival inflammation (21 , 24). It has been reported that the modification of salivary secretion in asthmatic individuals could negatively impact upon periodontal health. Saliva exerts a protective effect, and a decrease in salivary secretion could affect the interaction between bacterial and immune factors, causing the gingival tissues to become more vulnerable to inflammation in asthmatic people (6). In this respect, the drugs used to treat asthma and which have an effect upon salivary secretion could also impact upon the severity of periodontal disease (24). On the other hand, some studies have reported that inhaled corticosteroids can influence bone calcium metabolism and lead to a decrease in bone mineral density (26 , 27). Hanania et al. (26) observed that asthmatic patients who use drugs of this kind could experience suppressed adrenal gland function and a decrease in bone density over time. This association in turn could influence the onset and progression of periodontal disease, especially at high drug doses and in prolonged treatments (28). However, it must be noted that these observations correspond to adult patients, and that periodontal disease may become more manifest with advancing age (23); such data therefore cannot be compared with our own findings. Considering the severity of asthma, we found no significant differences in the level of oral hygiene between the children with mild asthma and those with moderate-severe asthma. Likewise, no differences were observed in terms of the amount of dental plaque and tartar, or in the gingival and periodontal disease indices. However, the patients with moderate-severe asthma showed a greater tendency to suffer bleeding on probing. In contrast to the above, Chakiri et al. (4), Arafa et al. (5) and Anandhan et al. (29) found the dental plaque and gingival indices to be significantly higher among patients with severe asthma. Likewise, according to Kumar et al. (8), dental plaque and gingivitis increase significantly with the severity of asthma. This could be because increased asthma severity implies an increase in the dose and frequency of administration of antiasthma drugs, resulting in a decrease in salivary flow. The consequent reduced salivary clearance capacity would in turn favor plaque accumulation. On the other hand, increased asthma severity would elevate the intensity of mouth breathing, giving rise to increased gingivitis (8). Thus, asthmatic children showed a greater presence of mouth breathing accompanied by greater gingivitis scores and bleeding on probing than the controls. No differences in oral hygiene level were observed between the two groups, however. Preventive programs should be applied in asthmatic children, with the promotion of a multidisciplinary approach for the management of asthma disease in order to prevent the development of oral tissue disorders. Regarding the limitations of our study, mention must be made of the fact that the oral examinations were made without blinding. In turn, collection of part of the information based on a questionnaire could have produced memory bias in the parents. Lastly, as this was a cross-sectional case-control study, no causal relationships could be established. Nevertheless, increased gingival vulnerability was observed in children with asthma than in children without asthma.

## Figures and Tables

**Table 1:** Description of the periodontal parameters by groups: results of the study model (ANOVA).

	Group	Mean	Standard deviation	F	p-value
Plaque index	Total	0.80	0.41		
	Control	0.80	0.47	0.72	0.396
	Asthma	0.79	0.36		
Oral hygiene index	Total	1.40	0.47		
	Control	1.38	0.55	0.13	0.710
	Asthma	1.42	0.41		
Gingival index	Total	1.43	0.30		
	Control	1.36	0.32	8.03	0.005*
	Asthma	1.48	0.27		
Bleeding index (%)	Total	61.21	17.45		
	Control	57.74	18.10	6.29	0.013*
	Asthma	63.76	16.58		
Community periodontal index	Total	1.34	0.31		
	Control	1.36	0.30	0.07	0.780
	Asthma	1.33	0.32		

*p<0.05; **p<0.01; ***p<0.001

**Table 2:** Description of the periodontal parameters by severity of asthma: results of the study model (ANOVA).

	Group	Mean	Standard deviation	F	p-value
Plaque index	Mild	0.81	0.37	0.83	0.364
	Moderate-severe	0.75	0.35		
Oral hygiene index	Mild	1.44	0.42	0.48	0.489
	Moderate-severe	1.39	0.41		
Gingival index	Mild	1.46	0.27	0.93	0.335
	Moderate-severe	1.52	0.28		
Bleeding index (%)	Mild	61.51	16.31	3.35	0.070
	Moderate-severe	68.07	16.46		
Community periodontal index	Mild	1.34	0.32	0.01	0.904
	Moderate-severe	1.31	0.31		

2

## Data Availability

The datasets used and/or analyzed during the current study are available from the corresponding author.
